# Swimming with the giant: coexistence patterns of a new redfin minnow *Pseudobarbus skeltoni* from a global biodiversity hot spot

**DOI:** 10.1002/ece3.2328

**Published:** 2016-09-15

**Authors:** Wilbert T. Kadye, Albert Chakona, Martine S. Jordaan

**Affiliations:** ^1^ Department of Ichthyology and Fisheries Science Rhodes University P.O. Box 94 Grahamstown 6140 South Africa; ^2^ South African Institute for Aquatic Biodiversity Private Bag 1015 Grahamstown 6140 South Africa; ^3^ CapeNature Scientific Services Private Bag X5014 Stellenbosch 7599 South Africa

**Keywords:** Freshwater fishes, habitat niche, morphological traits, niche overlap, trophic niche

## Abstract

Ecological niche theory predicts that coexistence is facilitated by resource partitioning mechanisms that are influenced by abiotic and biotic interactions. Alternative hypotheses suggest that under certain conditions, species may become phenotypically similar and functionally equivalent, which invokes the possibility of other mechanisms, such as habitat filtering processes. To test these hypotheses, we examined the coexistence of the giant redfin *Pseudobarbus skeltoni*, a newly described freshwater fish, together with its congener *Pseudobabus burchelli* and an anabantid *Sandelia capensis* by assessing their scenopoetic and bionomic patterns. We found high habitat and isotope niche overlaps between the two redfins, rendering niche partitioning a less plausible sole mechanism that drives their coexistence. By comparison, environment–trait relationships revealed differences in species–environment relationships, making habitat filtering and functional equivalence less likely alternatives. Based on *P. skeltoni*'s high habitat niche overlap with other species, and its large isotope niche width, we inferred the likelihood of differential resource utilization at trophic level as an alternative mechanism that distinguished it from its congener. In comparison, its congener *P. burchelli* appeared to have a relatively small trophic niche, suggesting that its trophic niche was more conserved despite being the most abundant species. By contrast, *S. capensis* was distinguished by occupying a higher trophic position and by having a trophic niche that had a low probability of overlapping onto those of redfins. Therefore, trophic niche partitioning appeared to influence the coexistence between *S. capensis* and redfins. This study suggests that coexistence of these fishes appears to be promoted by their differences in niche adaptation mechanisms that are probably shaped by historic evolutionary and ecological processes.

## Introduction

Ecological theory assumes that coexistence within communities is structured by an interplay between environmental and evolutionary processes (Holt 2001). In classic ecological theory, niche‐based mechanisms are considered to be central because they describe interspecific niche differentiation as a factor that facilitates resource partitioning among co‐occurring species (Leibold and McPeek 2006; Soberon and Nakamura 2009). From a niche perspective, environmental factors are assumed to provide a template that shapes interspecific differences in life history strategies, behavioral and functional traits (Southwood 1988; Diaz et al. 2001), whereas feeding interactions define the trophic role of a species within an ecosystem (Leibold 1995). Because species differ in their environmental requirements, trait‐based studies within and among communities provide a resolution through which assemblages and species niches are structured along environmental gradients (McGill et al. 2006; Cornwell and Ackerly 2009). Similarly, assessing the trophic role of a species provides an understanding of its interaction with other species from a food web perspective (Layman et al. 2007). Thus, niche theory elucidates deterministic processes that facilitate species coexistence and has been used to account for species diversity within ecosystems (Hutchinson 1961; Silvertown 2004), ecological and functional specialization of species within communities (Wright 2002) and phylogenetic niche conservatism among closely related species (Violle et al. 2011).

The applicability of niche theory is particularly appealing when examining the coexistence patterns of closely related and cryptic species (Wellborn and Cothran 2007). Both empirical and theoretical studies have shown that coexistence among closely related and cryptic species may be driven by phylogenetic niche conservatism that imposes strong interspecific competition, leading to resource partitioning (Violle et al. 2011; Maire et al. 2012). It is assumed that closely related species may coexist through niche partitioning along both biotic and abiotic axes (Kraft et al. 2015), which is likely to be favored when such species possess dissimilar traits that allow them to avoid competitive exclusion (MacArthur and Levins 1967; Pacala and Tilman 1994). Possessing different resource acquisition traits reduces the intensity of interspecific interactions and promotes complementary use of resources when closely related species co‐occur (Silvertown 2004; Carroll et al. 2011).

While niche differentiation is considered central in stabilizing interspecific interactions, closely related and cryptic species often exhibit close phenotypic similarities, thereby becoming functionally equivalent (McPeek and Gomulkiewicz 2005). This poses a challenge to foster niche‐based processes as the sole drivers to coexistence (Leibold and McPeek 2006). Although unequivocal evidence has been presented on the role of niche partitioning processes in promoting (or facilitating) coexistence of phenotypically similar species (Violle et al. 2011; Michalko and Pekar 2015), alternative mechanisms, such as habitat filtering, neutral processes and nonequilibrium dynamics, have been identified as potential drivers of species coexistence (Hubbell 2001; Chave 2004; McGill et al. 2006). Habitat filtering is assumed to favor selection, from a regional pool, of species that possess traits that are suitable for a particular environment (Keddy 1992; Diaz et al. 1998). This promotes evolution of converging functional traits and life history strategies that enable different species to adapt to most frequently encountered environments, allowing them to coexist with limited competitive exclusion (McGill et al. 2006). By imposing ecological filters that favor certain ecological traits for a given environment, habitat filtering excludes functionally dissimilar species because they cannot cope with local environmental conditions (Grime 1973; Mayfield and Levine 2010). Due to the contrasting outcomes of niche‐ and habitat‐based mechanisms, there is need for studies that explicitly test the relative roles of alternative ecological mechanisms to improve our understanding of the factors that underpin the coexistence of cryptic or closely related species.

Freshwater fishes of the cyprinid genus *Pseudobarbus* (commonly referred to as redfins) represent an appropriate model taxa for ecological research on the mechanisms that promote coexistence of closely related species. Fishes of this monophyletic genus are endemic to the Cape Fold Ecoregion (CFE) of South Africa, with one species restricted to the Lesotho Highlands (Skelton 1990, 2001). Some of the redfin species occur in sympatry within headwater tributaries of the Breede, Gouritz and Gamtoos river systems (Skelton 1990, 2001; Chakona and Swartz 2013). The co‐occurrence of closely related species of redfins in these headwater streams posits the likelihood of niche‐based processes as drivers of their coexistence. Niche‐based coexistence may occur due to the conservatism of either phylogenetic, functional or ecological traits, which may promote divergence in resource use by different species (Pyron et al. 2015). Nevertheless, the headwater streams within the CFE are considered to be harsh environments that are characterized by extremes of high flows and low water temperatures during the winter months, contrasted with dry conditions, low flows and high temperatures during the summer period (Dallas and Rivers‐Moore 2014). Because of these conditions, it is likely that other processes, such as habitat filtering (Carnicer et al. 2008; Mouchet et al. 2010), are also likely to influence the coexistence patterns. These headwater streams of Cape Fold Mountains are oligotrophic because the catchments that they drain are highly weathered and nutrient‐poor (de Moor and Day 2013). Furthermore, these streams are characterized by low habitat heterogeneity as they are dominated by coarse substratum (mainly cobbles, boulders, and bedrock), with little or no in‐stream macrophytes. It is therefore likely that under such extreme conditions, coexistence may be regulated by environment‐ and habitat‐driven factors, which may promote convergence of functional and ecological traits, thereby superceding the role of resource partitioning.

**Figure 1:**
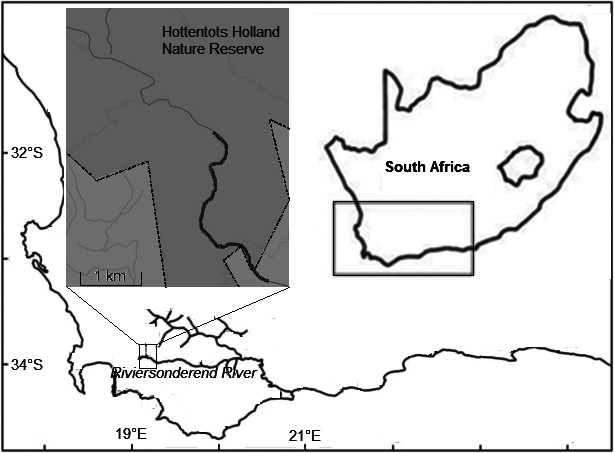
Map of the study area showing the sampled section of the Upper Riviersonderend River within the Hottentots Holland nature Reserve, Western Cape, South Africa.

To understand the relative importance of niche‐based resource partitioning and habitat filtering mechanisms in promoting species co‐existence, we examined the ecological patterns associated with the newly described giant redfin minnow *Pseudobarbus skeltoni* (Chakona and Swartz 2013) in the CFE. Currently, little is known about the basic ecology and the conservation status of this new species, apart from it having a restricted distribution within the Breede River system where it is known from only a few localities. *Pseudobarbus skeltoni* co‐occurs with two other species in the Upper Riviersonderend River, its congener *Pseudobabus burchelli* and an anabantid *Sandelia capensis*. The objective of this study was to compare habitat use and trophic ecology of these three co‐occurring species within an undisturbed 5 kilometer stretch of the Upper Riviersonderend (Fig. 1). Current conservation concerns on redfin species precluded protracted spatial and temporal sampling of these populations. This study therefore relied on short‐term sampling to jointly examine both scenopoetic and bionomic patterns. We hypothesized that if coexistence of these taxa is driven by niche‐based processes, these species would be expected to show a pattern of habitat and/or trophic niche differentiation and exhibit different morphological functional traits. Alternatively, if habitat filtering processes drive coexistence, the taxa are expected to show a pattern that is consistent with functionally equivalent species and exhibit similar morphological traits.

## Methods

### Study species

Fine‐scale geographic sampling approaches and recent phylogeographic studies have facilitated more accurate mapping of the distribution ranges of stream fishes in the CFE (Chakona and Swartz 2012; Chakona et al. 2013). This study focused on three co‐occurring taxa in the Upper Riviersonderend River: the recently described *P. skeltoni* (Chakona and Swartz 2013), the Breede lineage of *P. burchelli* (hereafter *P. burchelli* sp. “Breede”), and the Riviersonderend lineage of *S. capensis* (hereafter *S. capensis* sp. “Riviersonderend”) (Chakona et al. 2013). *Pseudobarbus skeltoni* is currently known from only three tributaries in the Breede River system, the Upper Riviersonderend and the Krom and Tierkloof Rivers in the Upper Breede (Chakona and Swartz 2013). *Pseudobarbus burchelli* sp. “Breede” occurs in three currently isolated river systems, the Breede, Duiwenhoks, and Goukou, whereas *S. capensis* sp. “Riviersonderend” occurs in the Riviersonderend, a major subcatchment of the Breede River system and the adjacent Palmiet River system (Chakona et al. 2013). Accurate assessment of the conservation statuses of these threatened taxa requires an understanding of their ecology and the processes that promote their persistence.

### Data collection

Sampling was conducted in March 2014 within an undisturbed 5 km stretch (between coordinates 34°02′49.1″S; 19°03′30.7″E upstream and 34°03′54.7″S; 19°04′51.4″E downstream) of the Upper Riviersonderend River (Fig. 1). *Pseudobarbus skeltoni*,* P. burchelli* sp. “Breede,” and *S. capensis* sp. “Riviersonderend” were the only fish species that occurred in the sampled section of the river. Due to the current conservation concerns on redfins within the CFE, sampling was conducted at a limited temporal and spatial scale. Fish were collected using nondestructive fyke nets (two‐panel D‐ring with 4‐mm mesh size) that were randomly positioned in pools and riffles within the sampled stream reach. A total of 37 sites were sampled over four consecutive days. The fyke nets were deployed at 1600 h and retrieved at 0800 h. Fish were identified to species, counted, and released. In addition, a total of 10 individuals of each fish species were collected for both stable isotope analysis and for measuring morphological traits, after which they were deposited as voucher specimens at the South African Institute for Aquatic Biodiversity (SAIAB) for future reference. The fish were euthanized by a lethal dose of clove oil (1 mL of clove oil/1 L of water). We characterized the microhabitat around each sampling site based on mean depth, habitat type, and dominant substratum. Mean depth was calculated from three points that were measured at each sampling site. These measurements were taken randomly along the fyke net. Habitat type was categorized as either riffle (<1 m mean depth) or pool (>1 m mean depth). Substratum composition was categorized based on a modified Wentworth scale (Bovee 1986) as sand (0.5–1 mm), gravel (10–60 mm), cobble (60–250 mm), boulder (250–1000 mm), and bedrock (>1000 mm). In addition, at each site, we measured physical and chemical parameters, including water temperature (°C), pH, conductivity (μS·cm^−1^), and total dissolved solids (TDS) (ppm) using a HANNA HI 98703 Combo meter.

To examine trophic niche relationships, samples of fish muscle tissue, macroinvertebrates, and plant matter were collected for stable isotope analysis. A sample of fish muscle tissue was taken above the lateral line in front of the dorsal fin. Macroinvertebrates were collected using a handheld scoop net (mesh size 250 μm). Sampling for macroinvertebrates was conducted by disturbing and washing‐off the animals from stones in riffles, and by repeatedly sweeping across vegetated habitats for 1 min in an area approximately 1 m^2^. We collected plant matter that included periphytic algae that were attached to substratum, and both emergent and submerged macrophytes. These were either hand‐picked or scrapped‐off from substratum using scalpel blades, after which the plant matter were thoroughly rinsed with river water. Samples for stable isotope analysis were collected randomly across all sampling sites. All samples for stable isotope analysis were kept in ice in the field and frozen until further analysis. In the laboratory, stable isotope samples were thawed and washed in distilled water. Macroinvertebrates were sorted and identified to family under a dissecting microscope at 10× magnification. Caddisflies (family Hydroptilidae) were detached from their cases prior to processing. All samples were oven‐dried at 60°C until to a constant weight was reached, after which they were ground to a fine powder. Samples were analyzed using a Europa Scientific 20–20 IRMS interfaced to an ANCA SL Elemental Analyser at the IsoEnvironmental Lab, Grahamstown, South Africa. Stable isotope ratios, δ^13^C and δ^15^N, were determined in parts per thousand (‰) relative to Vienna Pee Dee Belemnite and atmospheric nitrogen standards, respectively, and according to the formula: δ^13^C or δ^15^N = (*R*_sample_/*R*_standard_ − 1) × 1000, where *R* = ^13^C/^12^C or ^15^N/^14^N.

To determine the role of functional traits, we measured standard length (Appendix 1) and the morphological traits related to habitat use and swimming behavior (Table 1). Morphological measurements for functional traits followed Chakona and Swartz 2013; whereas swimming behavior traits were based on Webb's (1984) criteria. Standard length measurements showed that *P. skeltoni* was distinguished by having individuals attaining larger length compared to its congener *P. burchelli* sp. “Breede” that had relatively small‐sized individuals (Appendix 1). Quantitative traits included maximum body depth, body width, and caudal peduncle dimensions (Webb 1984). These were measured using digital Vernier calipers to the nearest 0.1 cm. To correct for potential allometric bias, measurements were adjusted based on Reist (1985) equation as follows: $M_{\mathrm{adj}}=\log Y-\beta \left(\log X-\log{\bar{X}}_{\mathrm{STL}}\right),$ where *M*_adj_ is the size transformed measurement, *Y* is the original unadjusted measurement, *β* is the allometric coefficient (the slope of the relationship of log *Y* against log *X*), *X* is the standard size measure of an individual, and ${\bar{X}}_{STL}$ is the mean of standard size measures across all individuals. Qualitative traits included mouth position and locomotor functional mechanism. Mouth position, which represent feeding behavior and habitat use, was described as being inferior for *P. burchelli* sp. “Breede” and terminal for *S. capensis* sp. “Riviersonderend” (Skelton 2001). By comparison, *P. skeltoni* exhibit an ontogenetic variability in this trait, with juveniles and subadults possessing inferior mouth, whereas adults have a terminal mouth (Chakona and Swartz 2013). Therefore, this species was recorded as having inferior or terminal mouth. Locomotor functional mechanism represents locomotion and habitat use behaviors (Webb 1984). It was described as being body‐caudal‐fin (BCF) periodic propulsion for both redfin species, and being BCF transient propulsion for *S. capensis* sp. “Riviersonderend.” BCF periodic propulsion is designed for sustained swimming and is typical of minnows that are considered to be generalist feeders (Webb 1984). By comparison, BCF transient propulsion is designed for rapid movements either to capture prey or evade predators and is characteristic of fishes with large body depth (Webb 1984).

**Table 1:** Morphological traits and their functional significance for *Pseudobarbus skeltoni*,* Pseudobabus burchelli* sp. “Breede,” and *Sandelia capensis* sp. “Riviersonderend” that were sampled in the upper Riviersonderend River, Western Cape, South Africa

Morphological trait	Trait category	Species		
		*Pseudobarbus skeltoni*	*Pseudobabus burchelli*	*Sandelia capensis*
Body depth	Locomotion, habitat use	26.8 ± 2.31 (3.6 ± 0.01)	25.2 ± 0.97 (3.0 ± 0.05)	32.0 ± 1.60 (3.3 ± 0.12)
Body width	Locomotion	18.4 ± 2.33 (5.1 ± 0.23)	16.5 ± 0.84 (3.9 ± 0.08)	19.0 ± 1.66 (4.2 ± 0.08)
Caudal peduncle length	Locomotion	22.3 ± 0.76 (5.0 ± 0.10)	25.6 ± 1.00 (4.2 ± 0.06)	14.0 ± 0.80 (3.6 ± 0.05)
Caudal peduncle depth	Locomotion	12.2 ± 0.49 (4.3 ± 0.03)	12.0 ± 0.60 (3.5 ± 0.05)	14.8 ± 0.65 (4.5 ± 0.04)
Mouth position	Feeding, habitat use	Inferior/terminal	Inferior	Terminal
Locomotor propulsion mechanism	Locomotion, habitat use	BCF periodic	BCF periodic	BCF transient

The measurements are means (and their associated standard deviations) that were expressed as proportion of standard length, and the values in parenthesis were the allometry‐adjusted measurements that were used in the analyses. Locomotor propulsion mechanisms were given as body‐caudal‐fin (BCF) periodic propulsion for both redfin species, and BCF transient propulsion for *Sandelia capensis* sp. “Riviersonderend.”

### Habitat niche overlap analysis

Streams offer a mosaic of microhabitats that influence resource use by fishes. Microhabitat availability influence several aspects of stream fishes, such as abundance, life history traits, resource use, and interspecific interactions (Copp 1992; Eros et al. 2003). For stream fishes, microhabitat availability can be quantified across multiple axes, including spatial distribution within micro‐ and meso‐habitats based on species occurrence, habitat association based on electivity data within defined microhabitats, and relative abundance based on count data along continuous gradients. In order to assess the hypothesis on habitat niche overlap (NO), we jointly examined different data types within a unified NO framework that emphasized local realized niches for different species (Geange et al. 2011). We analyzed spatial NO for the three species based on three microhabitat dimensions; habitat type, substratum type, and depth. NO and null models were calculated following the approach of Geange et al. (2011). This approach takes into account multiple niche axes, each being characterized by a different data type, and computes a unified NO. For the habitat type dimension, the response (occurrence of a species in either pools or riffles) was treated as binary data and was modeled based on Bernoulli distribution. NO between two species along this dimension was calculated as the joint probability of occurrence and absence along the resource axis. Statistical significance of NO was modeled based on probabilities of occurrence using randomization tests. For the substratum dimension, the association of a species to a particular substratum type was treated as electivity data. First, all sites were categorized based on the dominant substratum type to create a habitat availability vector. The availability vector of the substratum types was scaled to proportion based on the number of sampling sites. Second, for each species, a habitat use vector was created based on the proportional occurrence within the respective substratum categories. Manly's alpha indices were then used to calculate NO for the electivity data (Geange et al. 2011). For the depth dimension, the number of fish at each site was treated as count data. NO along the depth gradient was therefore modeled based on Poisson distribution by creating density distributions based on nonparametric maximum‐likelihood estimates (Norris and Pollock 1998). This produces Poisson distributions that are analogous to kernel density estimates, whereby NO between two species is calculated from the joint probabilities of the fitted distribution. A unified NO between species *i* and *j* for the mixed data sets was obtained by averaging NO from the different dimensions and was given as:

$${\text{NO}}_{i,j}=\frac{1}{T}\sum_{t=1}^{T}{\text{NO}}_{i,j,t}$$

where NO_*i*,*j*_ is the niche overlap value that ranges from 0, when distribution of two species is completely separate, to 1, when they completely coincide, and *T* is the number of dimensions (Geange et al. 2011). To test the statistical significance of NO between a pair of species, null model permutation tests were conducted to test whether mean NO was significantly lower than expected by chance. Statistical null distributions (*H*_0_ = no niche differentiation) were generated by computing pseudo‐values through random permutation of species data. We used 1000 permutations to test the significance of each microhabitat dimension and for the mean NO. Nonmetric multidimensional scaling (NMDS) was used to graphically visualize the results.

### Species–environment–trait relationship analysis

Because the sampling localities were contiguous, environmental variables were used to test the assumption of independence among sampling sites. This was based on covariance comparison of environmental variables using Moran's *I* and Geary's *C* randomization tests (Thioulouse et al. 1995). We used water chemistry and depth measurements for these comparisons. The results of this analysis revealed that the sampling sites were spatially autocorrelated (Appendix 2). In order to account for the lack of independence, generalized linear mixed models (GLMMs) were used to analyze fixed effects and spatially autocorrelated random effects (Dormann et al. 2007; Bolker et al. 2009). First, for all species, we used GLMMs to test the environment–trait relationship (Jamil et al. 2013). We used microhabitat factors (habitat type, depth, and substratum type) as environmental variables, and morphological characteristics (body depth, body width, caudal peduncle depth, caudal peduncle length, mouth position, and locomotor propulsion mechanism) as trait factors. The environment–trait relationship was tested as an interaction term in the model of the form: *y*_*ij*_ = *α*_0_ + *α*_1_ *X*_*i*_ + *β*_1_ *Z*_*j*_ + *β*_2_ *X*_*i*_ *Z*_*j*_ + *ɛ*_*αj*_ + *γ*_*i*_, where *α*_0_ is the intercept, *α*_*i*_ is the slope of the *j*th species with respect to environmental variable *X*_*i*_, *β*_1_ is the slope of the *j*th species with respect to trait factor *Z*_*j*_, and *β*_2_ represents the environment–trait interaction *X*_*i*_ *Z*_*j*_. The random effects of the model were specified for species (*ε*_*αj*_) and sites (*γ*_*i*_). Using fish counts (number of fish per fyke net for each species) as response, data were modeled using Poisson distribution and Laplace approximation for maximum‐likelihood estimation of parameters. For each environmental variable, we created multiple environment–trait relationship models. Each of these models was compared, using likelihood ratio tests, to a null model that comprised of species and spatially autocorrelated sites as random factors. The most optimal model was selected based on the lowest Akaike information criterion (AIC) values and highest Akaike weights (*w*_*i*_) (Burnham and Anderson 2002). To test the significance of environment–trait interaction, we compared the most optimal models to a null model (*β*_1_ = 0) that excluded the interaction term. The two models were compared using likelihood ratio tests. The contribution of the trait to the interspecies variance was compared using the estimates of the variances in the optimal and null models, given as:

$$c_{\beta }=1-\frac{{\hat{\sigma }}_{\beta }^{2}\left(\text{residual}\right)}{{\hat{\sigma }}_{\beta }^{2}\left(\text{total}\right)}$$

where ${\hat{\sigma }}_{\beta }^{2}\left(\text{residual}\right)$ is residual variance of the model with the interaction (optimal model), and ${\hat{\sigma }}_{\beta }^{2}\left(\text{total}\right)$ is residual variance of the model without interaction (null model) (Grosbois et al. 2009; Jamil et al. 2013). Second, for each species, we used GLMMs to investigate the statistical relationships between abundance and microhabitat variables. The response variable was Poisson distributed count data (number of fish per fyke net at each site). Predictor variables were mean depth (m), substratum type (sand, gravel, cobble, boulder, and bedrock), habitat type (pool or riffle), and their two‐way interactions (substratum × habitat), and sampling sites were included as the random variable. The statistical significance of the fixed effects was tested using Wald's *χ* ^2^ tests.

### Stable isotope analysis

To investigate the trophic niche structure of the study species, δ^13^C and δ^15^N were used to derive quantitative population metrics (Layman et al. 2007) for each species. The δ^13^C values were not adjusted for potential lipid‐accumulation bias (Post et al. 2007) because the C:N was less than 3.5 in all fish samples. Metrics were computed using the Stable Isotope Bayesian Ellipses in R (SIBER, Jackson et al. 2011). These metrics included: δ^13^C range (CR) that indicates trophic diversity at the base of a food web; δ^15^N range (NR) that provides information on trophic length; distance to centroid (CD), which is the average Euclidean distance of all components to the centroid giving an indication on average trophic diversity, and standard ellipse area (SEA that was expressed as ‰^2^) as a measure of trophic niche structure (Jackson et al. 2011). A small sample size‐corrected standard ellipse (SEA_c_) was used to compare trophic niche sizes. The uncertainty associated with each SEA was determined using Bayesian distributions (SEA_B_), thereby facilitating a comparison of trophic niche sizes between species. Two other metrics, mean nearest neighbor distance (MNND), and standard deviation of nearest neighbor distance (SDNND) provide information on trophic redundancy. The variability of each metric was estimated using nonparametric bootstrapping of the observed δ^13^C–δ^15^N data (Efron and Tibshirani 1993).

We determined the isotopic niche size for each species by calculating trophic niche region (*N*_R_) and compared the degree of trophic NO between species following the approach of Swanson et al. (2015). Trophic *N*_R_ is defined as a specific region within *n*‐dimensional isotopic space in which individuals of a particular species have the probability *α* of being found. In this study, we used *α* = 95%. Trophic NO was calculated as the probability that an individual from one species would be found in the *N*_R_ of another species (Swanson et al. 2015). Uncertainty associated with posterior distribution of both trophic *N*_R_ and NO was conducted within Bayesian framework.

The relative contribution of dietary sources assimilated by the three species was estimated using bivariate Bayesian isotopic mixing model, Stable Isotope Analysis in R package (SIAR, Parnell et al. 2010). The SIAR model uses a Markov Chain Monte Carlo simulations based on Dirichlet distribution to produce simulations of plausible values that show dietary proportions of potential prey to the consumers. The resulting probability density distributions, with the mean proportion together with the 50, 75, and 95% credibility intervals (the Bayesian analogue of a confidence interval), were used to compare contributions of different source groups. A total of nine samples for *P. skeltoni* and 10 samples for both *P. burchelli* sp. “Breede” and *S. capensis* sp. “Riviersonderend” were used to investigate the trophic niche patterns. The sources were analyzed in triplicate, and these included detritus, periphytic algae, macrophytes, and several macroinvertebrate families. For the macroinvertebrates, families in orders Odonata (Coenagrionidae and Libellulidae), Ephemeroptera (Baetidae, Caenidae, and Leptophlebiidae), Trichoptera (Hydropsychidae and Hydroptilidae), and Plectoptera were grouped because they were not statistically different (*k*‐nearest neighbor distance, randomization tests, *P > *0.05). Discrimination factors between sources and consumers were assumed to be 1 ± 0.5‰ (Δδ^13^C) and 3 ± 0.5‰ (Δδ^15^N) (McCutchan et al. 2003).

All analyses were conducted within R (R Core Development Team 2015). Habitat NO indices and environment–trait relationships were analyzed based on an R scripts provided by Geange et al. (2011) and Jamil et al. (2013), respectively. Within R, the following packages were used; vegan for NMDS, lme4 for GLMMs, siar and nicheRover for stable isotope analyses.

## Results

### Habitat NO

**Figure 2:**
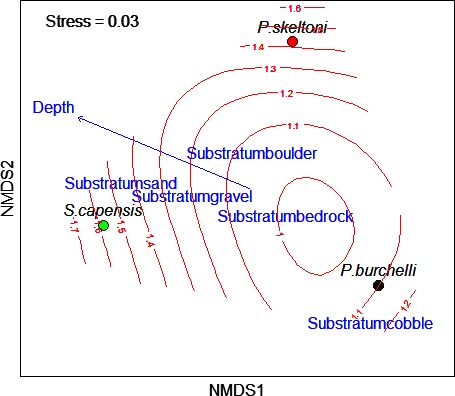
Interspecific similarities in unified realized niche overlaps among *Pseudobarbus skeltoni*,* Pseudobabus burchelli* sp. “Breede,” and *Sandelia capensis* sp. “Riviersonderend” assessed by nonmetric multidimensional scaling (NMDS) based on habitat type, depth and substratum type. The contours represent depth gradient based on generalized additive model (GAM) fits.

The sampled sites had clear water that was characterized by both low conductivity (mean ± SD = 27.59 ± 4.71 μS·cm^−1^) and low TDS (15.92 ± 2.63 ppm). Water pH ranged from slightly acidic (5.8) to neutral (7), and mean water temperature was 19.68 ± 1.06°C. A total of 695 fishes were captured. These included 63 individuals of *P. skeltoni*, 381 individuals of *P. burchelli* sp. “Breede,” and 259 individuals of *S. capensis* sp. “Riviersonderend.” All captured fishes (except those that were retained for stable isotope analysis [see above]) were released back to the river alive. The local realized habitat niche of *P. skeltoni* was found to overlap with that of both *P. burchelli* sp. “Breede” (mean NO = 0.94 ± 0.09, *P > *0.05) and *S. capensis* sp. “Riviersonderend” (NO = 0.97 ± 0.05, *P > *0.05). Similarly, there was no significant differences in local realized habitat niches between *P. burchelli* sp. “Breede” and *S. capensis* (mean NO = 0.92 ± 0.13, *P > *0.05). This indicated that all three fishes occurred within similar habitat conditions. Nevertheless, the spatial niche patterns based on NMDS analysis showed that *S. capensis* occurred in relatively deeper habitats than *P. skeltoni* and *P. burchelli* sp. “Breede” (Fig. 2).

### Environment–trait relationship

**Figure 3:**
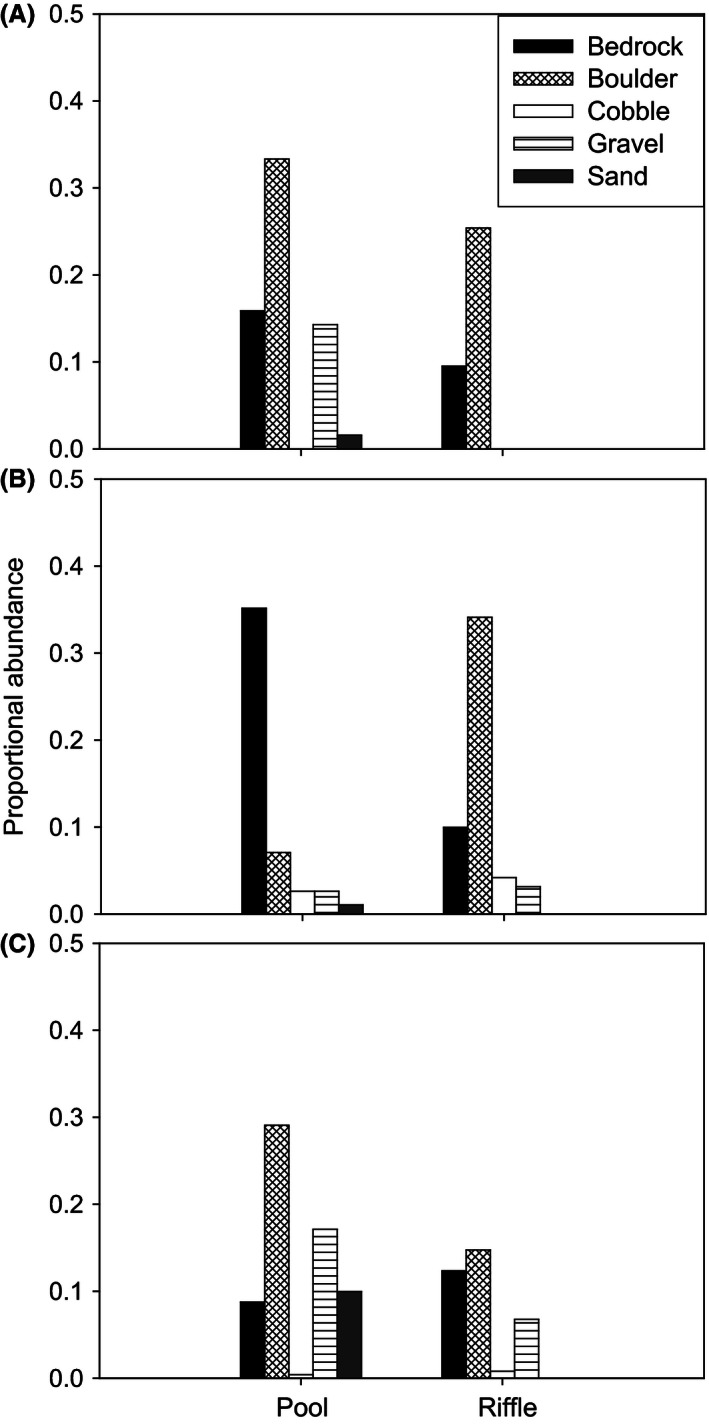
Proportional abundances of *Pseudobarbus skeltoni* (A), *Pseudobabus burchelli* sp. “Breede” (B), and *Sandelia capensis* sp. “Riviersonderend” (C) within pool and riffles in relation to different substratum types.

Two morphological traits, mouth position and caudal peduncle depth, were most influential in explaining environment–trait relationship (Table 2). Trait–environment interaction models showed that morphological trait mouth position accounted between 60 and 90%, whereas caudal peduncle depth accounted between 20 and 70% of species response to habitat variables. Based on the GLMM, species with inferior mouth were most abundant in riffles (riffle × inferior mouth, *β*_2_ = 0.68, *z* value = 2.43, *P = *0.02) and decreased in abundance with increasing water depth (depth × inferior mouth, *β*_2_ = −0.60, *z* value = −2.39, *P = *0.02). This pattern was illustrated by the distribution of *P. burchelli* sp. “Breede” whose abundance was significantly influenced by depth (GLMM, $\chi_{1}^{2}$ = 7.99, *P = *0.004). Although this species was ubiquitous and was captured in all substratum categories, it was most abundant in riffles (Fig. 3). In contrast to mouth position, species with large caudal peduncle depth were less abundant in riffles (riffle × caudal peduncle depth, *β*_2_ = −0.69, *z* value = −4.02, *P < *0.01) and were more abundant with increasing depth (depth × caudal peduncle depth, *β*_2_ = 0.84, *z* value = 4.48, *P < *0.01) at sites with either gravel (gravel × caudal peduncle depth, *β*_2_ = 1.95, *z* value = 5.63, *P < *0.01) or boulder (boulder × caudal peduncle depth, *β*_2_ = 0.87, *z* value = 3.74, *P < *0.01) substratum. This pattern was illustrated by the distribution of *S. capensis* sp. “Riviersonderend” and *P. skeltoni* that both had relatively large caudal peduncle depth. Abundances of *S. capensis* sp. “Riviersonderend” differed significantly among substratum types (GLMM, $\chi_{4}^{2}$ = 12.99, *P = *0.01), with this species being most abundant on boulder and gravel substratum within both pools and riffles (Fig. 3). By comparison, abundances of *P. skeltoni* differed significantly between habitat type (GLMM, $\chi_{1}^{2}$ = 6.33, *P = *0.01), depth (GLMM, $\chi_{1}^{2}$ = 6.12, *P = *0.01), and substratum types (GLMM, $\chi_{4}^{2}$ = 17.94, *P = *0.01). This species was most abundant in pools that had boulder and gravel substratum (Fig. 3).

**Table 2:** Summary of environment and trait interaction models explaining the abundances of *Pseudobarbus skeltoni*,* Pseudobabus burchelli* sp. “Breede,” and *Sandelia capensis* sp. “Riviersonderend” in the upper Riviersonderend River, Western Cape

Model	df	AIC	Log likelihood	Deviance	*χ* ^2^	*P*
Habitat × caudal peduncle depth	8	870.49	−427.25	854.49	5.38	0.02
Habitat × mouth position	10	861.28	−420.64	841.28	6.06	0.05
Depth × caudal peduncle depth	8	840.93	−412.47	824.93	5.27	0.02
Depth × mouth position	10	831.54	−405.77	811.54	8.14	0.02
Substrate × caudal peduncle depth	26	801.98	−374.99	749.98	9.85	0.04
Substrate × mouth position	31	798.15	−368.07	736.15	16.74	0.03

### Trophic niches

**Figure 4:**
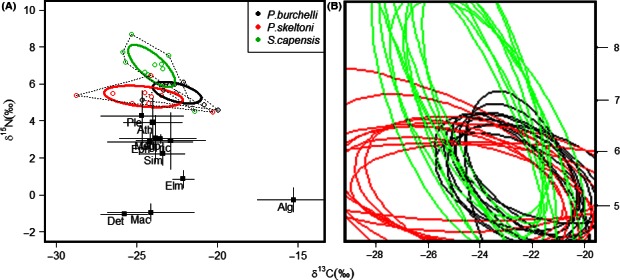
Biplots of δ^13^C and δ^15^N isotope data indicating food web characteristics of *Pseudobarbus skeltoni*,* Pseudobabus burchelli* sp. “Breede,” and *Sandelia capensis* sp. “Riviersonderend.” Panel a displays standard ellipse areas (SEA_c_) representing sample size‐corrected isotopic niche space (solid ellipses) and the convex hulls (dashed polygons) of the three fishes, and the mean δ^13^C or δ^15^N values of the potential food sources (black squares) and their associated standard deviations. The potential food sources included macroinvertebrate groups such as Plecoptera (Ple), Athericidae (Ath), Ephemeroptera (Eph), Megaloptera (Meg), Odonata (Odo), Trichoptera (Tri), Simuliidae (Sim), and Elmidae (Elm). Basal food sources included detritus (Det), macrophytes (Mac), and periphytic algae (Alg). Panel b displays ten random elliptical projections of trophic niche regions (NR) for each species defined by stable isotope values of δ^13^C and δ^15^N.

**Figure 5:**
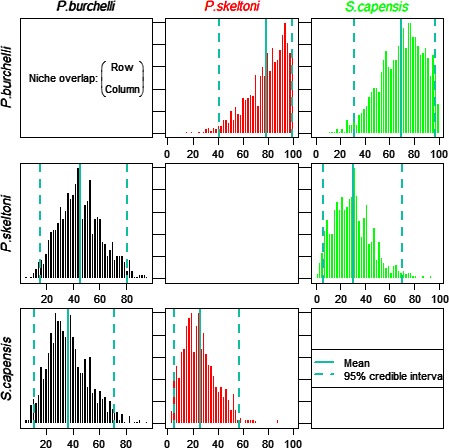
Comparison of posterior distribution of probabilistic trophic niche overlap for specified niche regions of *Pseudobarbus skeltoni*,* Pseudobabus burchelli* sp. “Breede,” and *Sandelia capensis* sp. “Riviersonderend.” Probabilities of niche overlap (mean and 95% credibility intervals) are specified as the overlap of species A (rows) onto the niche of species B (columns).

Stable isotope analysis revealed that *P. skeltoni* occupied a lower trophic position than other fishes (Fig. 4A). This species had the largest isotope niche size (SEA_c_ = 4.17‰^2^), which was consistent with the probabilistic estimates of its *N*_R_ (Fig. 4B), and was characterized by a wide breadth in carbon sources (CR = 8.46‰), high trophic diversity (CD = 1.64‰) an intermediate trophic range (NR = 1.97‰), and low trophic redundancy (MNND = 1.20‰, SDNND = 1.00‰). This showed that *P. skeltoni* utilized a wider range of food sources than other fishes. By comparison, its congener *P. burchelli* sp. “Breede” had the smallest isotope niche size (SEA_c_ = 2.36‰^2^), a pattern that was corroborated by probabilistic estimates of its *N*_R_ (Fig. 4B). This species exhibited a narrow breadth in both carbon sources (CR = 4.67‰) and trophic range (NR = 1.50‰). Furthermore, *P. burchelli* sp. “Breede” showed the lowest trophic diversity (CD = 1.16‰) and highest trophic redundancy (MNND = 0.61‰, SDNND = 0.39‰), indicating that it had a low degree of diversity in its food sources compared to other fishes. In contrast to the two redfins, *S. capensis* sp. “Riviersonderend” occupied a higher trophic position (Fig. 4A). Consistent with probabilistic estimates of its *N*_R_, this species was characterized by intermediate isotope niche size (SEA_c_ =3.56‰^2^), trophic diversity (CD = 1.32‰) and trophic redundancy (MNND = 0.75‰, SDNND = 0.85‰), and a narrow breadth in carbon sources (CR = 4.47‰). It was, nevertheless, distinguished by having the widest breadth in trophic range (NR = 4.20‰), indicating that it utilized a wider range in trophic links than other fishes. We found that there was a low probability of *P. skeltoni* overlapping onto the trophic niches of both *P. burchelli* sp. “Breede” (overlap probability = 46%) and *S. capensis* sp. “Riviersonderend” (overlap probability = 30%) (Fig. 5). By contrast, *P. burchelli* sp. “Breede” was found to have a high probability of overlapping onto trophic niches of *P. skeltoni* (overlap probability = 78%) and *S. capensis* sp. “Riviersonderend” (overlap probability = 69%). In comparison, *S. capensis* sp. “Riviersonderend” was found to have low probability (<48%) of overlapping onto the trophic niches of both redfin species.

**Figure 6:**
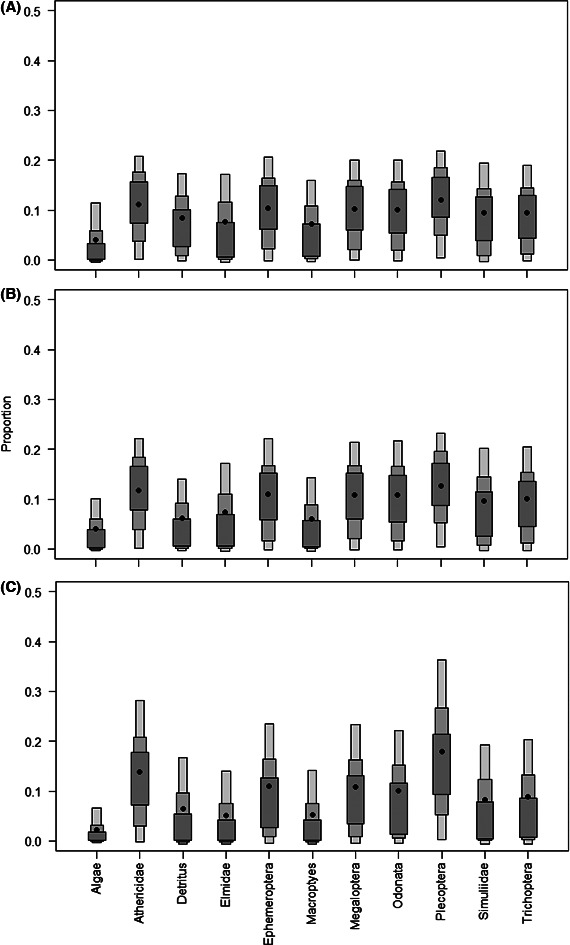
The estimated proportional source contributions of potential prey to the diets of *Pseudobarbus skeltoni* (A), *Pseudobabus burchelli* sp. “Breede” (B), and *Sandelia capensis* sp. “Riviersonderend” (C). Density estimates for the dietary source contributions were based on Bayesian inference, and the box plots for indicate 50, 75, and 95% credibility interval and the black dot indicates the mean.

Stable Isotope Analysis in R package mixing models indicated that both *P. skeltoni* and *P. burchelli* sp. “Breede” had diverse food sources, with macroinvertebrates being more important than plant matter (Fig. 6). Nonetheless, few prey sources, namely Athericidae, Ephemeroptera, Megaloptera, Odonata, and Plecoptera contributed >10% (95% credibility range = 0–22%) each to *P. skeltoni*'s diet. Similarly, these prey sources, together with Simuliidae, contributed >10% (95% credibility range = 0–23%) each to the diet of *P. burchelli* sp. “Breede.” By comparison, *S. capensis* sp. “Riviersonderend” diet comprised mostly of Plecoptera (median = 18%, 95% credibility range = 0–36%) and Athericidae (median = 14%, 95% credibility range = 0–28%). However, other prey sources, including Ephemeroptera, Megaloptera, and Odonata, were also important as they contributed >10% to its diet.

## Discussion

Based on the high habitat and isotopic trophic NO between *P. skeltoni* and its congener *P. burchelli* sp. “Breede,” the hypothesis of niche partitioning as the sole driver to their coexistence was rejected. The results indicated that there was no clear niche partitioning, either at habitat or trophic scale, between the two redfins. Theory suggests that in the absence of clear niche partitioning, habitat filtering processes are likely to drive coexistence due to convergence toward optimum traits that equilibrate interspecific interactions among species, thereby making such species functionally equivalent (Grime 2006; Carroll et al. 2011). However, our results on environment–trait relationships revealed that the two redfins exhibited interspecific differences in the role of morphological traits that influenced their microhabitat associations. Specifically, we found that due to its inferior mouth, *P. burchelli* sp. “Breede” exhibited high propensity toward riffles, whereas species with terminal mouth appeared to be most abundant in deeper habitats. By comparison, for *P. skeltoni*, we inferred that due to its possession of inferior and terminal mouth positions for juveniles and adults, respectively, this species was most likely to exhibit intraspecific difference in both habitat use and prey diversity. During this study, *P. skeltoni* juveniles and subadults were captured in shallow habitats together with *P. burchelli* sp. “Breede,” whereas adults were most abundant in deep habitats. Thus, habitat filtering and functional equivalence hypothesis appeared inadequate alternative mechanisms to explain the coexistence of the two redfins.

Results of the present study indicated that *P. skeltoni* had a large trophic niche that overlapped and encompassed that of *P. burchelli* sp. “Breede.” The large trophic niche for *P. skeltoni* suggests that it occupied broad ecological niches This suggests the two redfins differed in their resource utilization patterns from a trophic niche perspective, which may help to reduce the intensity of interspecific competition. Some studies have shown that populations or species that occupy larger ecological niches usually exhibit greater intraspecific phenotypic variation compared to those occupying smaller niches (Van Valen 1965). Intraspecific variability would manifest in different forms, including genetic variation, sexual dimorphism, and ontogenetic change (Galeotti and Rubolini 2004; Meiri et al. 2005). Species that have large ecological niches are assumed to be driven by eco‐evolutionary process that evolved to allow such species to utilize a wide diversity of resources in order to minimize intraspecific competition (Bolnick et al. 2007). For *P. skeltoni*, we inferred that high phenotypic variability was a factor that would potentially influence this species to utilize a broad range of resources. This is because not only does this species grow larger than its congener but also it is distinguished by having variable morphological characters, such as inferior mouth in juveniles and subadults, and terminal mouth in adults (Chakona and Swartz 2013). This posits the likelihood of this species having differential habitat use and feeding patterns during different life stages. We postulate that such a phenomena could have been shaped by past evolutionary processes associated with the speciation of this species. Redfins are considered to have evolved in allopatry through vicariance processes that led to the isolation of different lineages, with the headwaters and tributaries of the Breede River system acting as possible refugia for the isolated populations (Chakona et al. 2013). It is likely that isolation of *P. skeltoni* may have led to ecological release from interspecific competition, thereby facilitating niche expansion that manifest in its wide isotopic trophic niche, its propensity to utilize both deep and shallow habitats, and its high habitat NO with other species that was observed in this study. In contrast, its congener *P. burchelli* sp. “Breede,” which exhibited less morphological variability, appeared to have a more conserved isotopic trophic niche and had high preference toward shallow habitats despite it being the most abundant species.

The large trophic niche size that was observed for *P. skeltoni* suggests that it had a broader dietary spectrum compared to its congener. Despite these differences, the two redfins both showed high trophic diversity that was characterized by absence of dominant prey and relatively similar dietary proportions. Based on the isotope mixing model, macroinvertebrates appeared to be the most important prey for the redfins. This appeared consistent with other studies that have shown macroinvertebrates as being the most important prey for redfins (Whitehead et al. 2007). Other studies based on stomach contents have, nonetheless, indicated that their high gut to length ratio suggest a herbivorous feeding habit (Skelton 1980), and they have been found to consume both plant and detrital matter (Cambray and Stuart 1985). The low proportional abundance of plant matter in redfin diets that was inferred by stable isotope analysis may suggest that either these basal food sources were less important or the availability of this food source was determined by environmental and abiotic factors that influence primary productivity in these mountain streams. These habitats appear to be structurally simple and are characterized by low primary production because the rivers are oligotrophic (de Moor and Day 2013). Furthermore, these mountain streams receive little allochthonous organic input from the fynbos vegetation that dominate the upper catchments, thereby supporting low biomass of secondary productivity (de Moor and Day 2013). This probably promotes trophic diversification, as was observed for the two redfins. Overall, these studies suggest flexibility in the feeding habits of redfins, which is probably a requisite adaptive trait to maintain viable populations within the CFE mountain stream that are subject to the influence of proximate environmental factors, such as high seasonal changes in flow and low nutrients, which are likely to influence resource availability.

The lack of clear differentiation in resource utilization between the two redfins suggests that their coexistence could be driven by other mechanisms such as temporal dynamics and potential differences in life history strategies. A study by Cambray (1994) revealed that differences in both life history strategies and spawning behaviors were the likely mechanisms that facilitated the coexistence of two closely related redfin minnows, *Pseudobarbus asper* and *Pseudobarbus afer* in the Gamtoos River system. During this study, *P. skeltoni* was found in relatively low abundance compared to its congener *P. burchelli* sp. “Breede” that occurred in high abundance. Although there is no information on the life history pattern of *P. skeltoni*, previous studies on the breeding behavior of *P. burchelli* within the Breede River have shown that it has a protracted spawning period from September to February (Cambray and Stuart 1985). Such a breeding behavior most probably represent spawning patterns of the different lineages of the *P. burchelli* species complex and possibly included the recently described giant redfin *P. skeltoni* (Swartz et al. 2009; Chakona and Swartz 2013). Evidence from previous surveys on *P. skeltoni* suggests that this species aggregate in shallow pools and riffles in November and December, possibly to spawn. Elsewhere within the CFE, differences in both life history strategies and spawning behaviors have been observed between two closely related redfin minnows, *P. asper* and *P. afer*, and have been suggested as the mechanism that facilitates their coexistence and adaptations to varying environmental conditions (Cambray 1994).

In contrast to the redfins, resource use patterns by *S. capensis* sp. “Riviersonderend” appeared to be influenced by both environment–trait relationship and trophic niche partitioning. Due to its small caudal peduncle, *S. capensis* sp. “Riviersonderend” showed propensity to use deeper habitats compared to the redfins. Furthermore, from a trophic niche perspective, based on stable isotope analysis, this species occupied a higher trophic position, and its trophic niche had a relatively low probability of overlapping with those of the redfins. Our study support findings of previous studies that have described this species as a predator that feeds on aquatic insects and small fish (Siegfried 1963). Previous studies have also indicated that *S. capensis* tolerates a wide range of physical and chemical water conditions (Harrison 1952). However, molecular evidence has shown that *S. capensis* is a species complex that has probably been subjected to similar vicariance processes and postspeciation dispersal mechanisms that have shaped the evolution of different lineages as the redfins (Chakona et al. 2013). Nevertheless, this study suggests that, at metacommunity level, this lineage of *S. capensis* was perhaps influenced by niche partitioning mechanisms through trophic niche segregation that enabled it to co‐occur with the redfins.

To conclude, this study suggests that coexistence of these fishes appears to be promoted by their different niche adaptation mechanisms that are probably shaped by historic evolutionary and ecological processes. For the closely related redfins, differences in niche adaptations appear to be driven by functional differences in morphological traits. In particular, the morphological variability in the mouth position of giant redfin *P. skeltoni* suggests the potential for intraspecific differences in habitat association and high trophic diversity compared to its congener *P. burchelli* sp. “Breede.” Both these patterns were illustrated by *P. skeltoni*'s high habitat NO with its congener and its larger isotopic trophic niche breadth. In contrast, trophic niche differences appeared to be most important in distinguishing the redfins from *S. capensis*. These findings have important implications on the conservation of these species. Currently, most redfins within the CFE are threatened, mainly as a result of their susceptibility to predation by non‐native piscivores that include bass (*Micropterus* spp.), rainbow trout (*Onchorhynchus mykiss*), and sharptooth catfish (*Clarias gariepinus*) that have established viable populations in this region (Cambray and Stuart 1985; Cambray 2003; Woodford et al. 2005; Tweddle et al. 2009; Shelton et al. 2015). Although *P. skeltoni* is a recent description, historical distribution records and molecular evidence suggest that it was once widespread within the Breede River and its major tributaries, and probably occurred within main stem habitats (Chakona and Swartz 2013). Its current distribution appears to be a consequence of anthropogenic activities, particularly invasion impacts. This study therefore provides an insight on its basic ecology to inform future conservation of this species.

## Conflict of Interest

None declared.

## Appendix 2 Moran's / and Geary's *C* randomization tests of environmental variables that were sampled across all sites indicating the observed values (Obs), their standard deviation (Std. obs), and significance level (*P*‐value). The tests were based on 1000 Monte Carlo randomization simulations.

**Table:**

Test	Obs	Std. obs	*P*‐value
Temperature	0.28	3.36	0.003
pH	0.37	4.39	0.001
Total dissolved solids	0.25	3.07	0.003
Conductivity	0.23	2.92	0.006
Depth	0.32	4.06	0.002
